# Cranial Morphology of the Brachystelechid ‘Microsaur’ *Quasicaecilia texana* Carroll Provides New Insights into the Diversity and Evolution of Braincase Morphology in Recumbirostran ‘Microsaurs’

**DOI:** 10.1371/journal.pone.0130359

**Published:** 2015-06-24

**Authors:** Jason D. Pardo, Matt Szostakiwskyj, Jason S. Anderson

**Affiliations:** 1 Department of Comparative Biology and Experimental Medicine, Faculty of Veterinary Medicine, University of Calgary, Calgary, Alberta, Canada; 2 Department of Biological Sciences, University of Calgary, Calgary, Alberta, Canada; Raymond M. Alf Museum of Paleontology, UNITED STATES

## Abstract

Recumbirostran ‘microsaurs,’ a group of early tetrapods from the Late Carboniferous and Early Permian, are the earliest known example of adaptation to head-first burrowing in the tetrapod fossil record. However, understanding of the diversity of fossorial adaptation within the Recumbirostra has been hindered by poor anatomical knowledge of the more divergent forms within the group. Here we report the results of μCT study of *Quasicaecilia texana*, a poorly-known recumbirostran with a unique, broad, shovel-like snout. The organization of the skull roof and braincase of *Quasicaecilia* is found to be more in line with that of other recumbirostrans than previously described, despite differences in overall shape. The braincase is found to be broadly comparable to *Carrolla craddocki*, with a large presphenoid that encompasses much of the interorbital septum and the columella ethmoidalis, and a single compound ossification encompassing the sphenoid, otic, and occipital regions. The recumbirostran braincase conserves general structure and topology of braincase regions and cranial nerve foramina, but it is highly variable in the number of ossifications and their extent, likely associated with the reliance on braincase ossifications to resist compression during sediment compaction and mechanical manipulation by epaxial and hypaxial musculature. Expansion of the deep ventral neck musculature in *Quasicaecilia*, autapomorphic among recumbirostrans, may reflect unique biomechanical function, and underscores the importance of future attention to the role of the cervical musculature in contextualizing the origin and evolution of fossoriality in recumbirostrans.

## Introduction

The earliest tetrapods to show morphological adaptations to headfirst burrowing are the recumbirostrans, a group of tuditanomorph ‘microsaurs’ for which monophyly has been reasonably established [1]. Recumbirostrans are small tetrapods known from the Permocarboniferous characterized by their reduced complement of dermal skull bones, a unique atlanto-occipital joint, a shovel-like snout, and recumbent premaxilla. As currently constituted, Recumbirostra includes the pantylids, gymnarthrids, ostodolepids, brachystelechids, and, in some analyses, lysorophians [1–6]. Although previously considered by some workers to be possible ancestors of lissamphibians [4–6] or of caecilians specifically [1, 2, 7, 8], recent restudy of the morphology of recumbirostrans [9, 10] and caecilians [11, 12]) suggests that this is likely not the case, and that recumbirostrans may exhibit more morphology in common with amniotes than with lissamphibians.

Head-first burrowing, where the skull itself is employed as a locomotory organ to displace or compact soil, is generally achieved via conspicuous morphological adaptations to the musculoskeletal system of the head and neck [13, 14]. This novel use of the skull imposes a number of important functional constraints: the skull must resist forces imposed by the soil compression phase of the excavation cycle, the cross-sectional area of the skull must be minimized in order to reduce the amount of soil that must be displaced, the cervical musculature must be expanded in order to power the digging stroke, and the jaw must be modified to permit feeding in confined spaces. These functional constraints have driven high levels of convergence between diverse headfirst burrowing lineages [13–20], with some variation reflecting burrowing mode [13, 14] and substrate type [20]. Gross morphology consistent with that seen in modern headfirst burrowers has been identified in ostodolepid recumbirostrans for some time [21], but it is only recently that adaptations consistent with headfirst burrowing have been more broadly recognized within recumbirostrans in general [9, 10, 22]. Micro-computed x-ray tomography (μCT), especially, has permitted virtual dissection of the internal structures of the recumbirostran skull and identification of braincase morphology indicative of headfirst burrowing even in species with generalized gross morphology [10, 22]. However, this effort has largely been limited to identification of morphology consistent with headfirst burrowing rather than variation in skull shape that may indicate variation in burrowing mode or substrate preference among recumbirostrans.

The recumbirostran *Quasicaecilia texana* is unique among recumbirostrans in having a broad, plow-like snout [23], similar in some ways to ‘shovel-headed’ amphisbaenids, and presents an opportunity to understand morphological, and ultimately functional, specialization within a recumbirostran. *Q*. *texana* belongs to the Brachystelechidae, a group of miniaturized recumbirostrans with highly divergent crania. Other brachystelechids have a relatively conservative, ovoid skull [9, 24, 25], and none approach the condition seen in *Q*. *texana*. Comparability between these taxa and *Q*. *texana* is limited due to ambiguities in the original description of *Q*. *texana* and in the presence of bone and matrix obscuring much of the internal morphology of the skull. The present study rectifies this deficiency.

We scanned the type and only specimen of *Quasicaecilia texana* using μCT. This imaging modality allows us to visualize the braincase and other internal structures of the skull, and to better understand how the morphology of *Quasicaecilia* compares with the morphology of other recumbirostrans. We present here the results of our study of *Quasicaecilia texana*, including detailed description of the neurocranium, and provide a new reconstruction of the skull of this taxon, although a consideration of the phylogenetic implications will be reserved for a larger scale effort to be published elsewhere.

## Materials and Methods

We studied the holotype and only specimen of *Quasicaecilia texana*, USNM 22079. The specimen is permanently kept in the vertebrate paleontology collections of the National Museum of Natural History (USNM), Smithsonian Institute, Washington, District of Columbia, USA. For comparison, we also studied: the holotype of *Huskerpeton englehorni* [22], UNSM 32144, permanently held in the vertebrate paleontology collections of the University of Nebraska State Museum (UNSM), Lincoln, Nebraska, USA; the holotype of *Dvellecanus carrolli* [10], UCMP 202940 (previously UCLA-VP 2940), permanently held in the vertebrate paleontology collections of the University of California Museum of Paleontology (UCMP), Berkeley, California, USA; a skull attributed to *Brachydectes newberryi*, KUVP 49541, permanently kept in the vertebrate paleontology collections of the University of Kansas Museum of Natural History (KUVP), Lawrence, Kansas, USA; and the holotype skull of *Carrolla craddocki* [9], (TMM 40031–54), permanently housed in the vertebrate paleontology collections of the Texas Memorial Museum (TMM), Austin, Texas, USA. No permits were required for the described study, which complied with all relevant regulations.

The type specimen of *Quasicaecilia texana* was scanned at the University of Texas CT Facility (UTCT, Austin, Texas) 6 May, 2009. The skull was scanned at 210 kV and 0.13 mA. Interslice spacing was one line at 0.01812 mm and the field of reconstruction was 17 mm. The resolution of the resulting images is 1024 px by 1024 px, with 827 images in the transverse plane. The complete stack of 8-bit jpg images is permanently archived on FigShare (http://dx.doi.org/10.6084/m9.figshare.1409582).

The holotype specimen of *Huskerpeton englehorni* (UNSM 32144) was used for comparison. This specimen (and these CT data) has been described previously in detail [22]. The scan was performed at UTCT on 4 May, 2009. The skull was scanned at 80 kV and 4W. The resolution of the resulting images is 1024 px by 1024 px with 865 images in the transverse plane. Voxels are 27.737 μm.

The holotype specimen of *Dvellecanus carrolli*, UCMP 202940, was used for comparison. This specimen and CT data, are described in detail elsewhere [10]. Scanning was performed at the University of Calgary (Anderson Lab, McCaig Bone and Joint Institute) using a Skyscan 1173 on 24 September, 2013. The skull was scanned at 77 kV and 0.084 mA. The resolution of the resulting images is 1120 px by 1120 px, with 665 images in the transverse plane. Voxels are 21.30 μm. The scan stack was subsequently downsampled in ImageJ 1.46r to 181 images in the transverse plane, and cropped to a resolution of 536 px by 350 px.

The skull of *Brachydectes newberryi*, KUVP 49541, was used for comparison. This specimen and CT data will be described in detail elsewhere. The skull was scanned at the University of Calgary (Anderson Lab, McCaig Bone and Joint Institute) using a Skyscan 1173 on 24 July, 2012. The scan was scanned at 100 kV and 0.060 mA. The resolution of the resulting images is 1120 px by 1120 px, with 436 images in the transverse plane. The voxel size is 38.91 μm. The scan stack was subsequently downsampled to 397 images in the transverse plane, cropped to a resolution of 453 px by 668 px.

The skull of *Carrolla craddocki*, TMM 40031–54, was used for comparison. This specimen (and these CT data), has been described in detail elsewhere [9]. The skull was scanned at UTCT on 18 September 2007. The skull was scanned at 200 kV and 0.10 mA. Interslice spacing was two lines at 0.03824 mm, interpixel spacing was 0.018 mm, and field of reconstruction was 18 mm. The resolution of the resulting images was 1024 px by 1024 px, with 518 images in the transverse plane.

Image stacks were imported into Amira 5.4 (VGS, Burlington, MA, USA) for visualization. We used the LabelField module to assign voxels to three-dimensional bone volumes manually using the brush tool and threshold mask function, and then generated three-dimensional surface models based on these volumes with the SurfaceGen module. A complete surface model of the segmented skull is permanently available on FigShare (http://dx.doi.org/10.6084/m9.figshare.1409581).

### Neurocranial Morphology and Nomenclature in Recumbirostrans

Recumbirostrans, by virtue of having a more heavily-ossified neurocranium than many other early tetrapods, often preserve morphology that is not directly comparable to the ossifications found in other archaic tetrapods, modern lissamphibians, and amniotes, but is directly comparable to a cartilaginous template that is broadly conserved across tetrapods. Some ossifications are expanded to encompass regions of the braincase that generally remain cartilaginous in most early tetrapods (e.g. the ‘pleurosphenoid’ of recumbirostrans) or have coalesced into compound bones (e.g. the otoccipital of *Carrolla craddocki* [9]). This has led to a complex and problematic terminology for recumbirostran braincase ossifications that complicates both assessment of the homologies of recumbirostran braincase ossifications as well as study of the relationships of recumbirostrans to other early tetrapods. The absence of a more inclusive anatomical framework to which the highly ossified recumbirostran braincase can be compared has led to circularity in the identification of bone homologies (e.g. the supraoccipital, ‘pleurosphenoid’ and orbitosphenoids) and the phylogenetic topologies inferred from these statements of bone homology [2, 4–10, 21, 22], and has limited the addition of the rich record of braincase morphology to phylogenetic studies of early tetrapod evolution.

In order to avoid these issues, and to improve the overall comparability of the recumbirostran braincase to other early tetrapods, we have chosen instead to describe the braincase using nomenclature associated with cartilaginous neurocranial structures. This approach is supported by the fact that the structure of the tetrapod neurocranium is generally highly conserved across diverse taxa, permitting identification of homology of neurocranial structure even when the homology of specific ossifications is under debate. Because ossification of the braincase in osteichthyans (including tetrapods) occurs endochondrally, the assumption that bony structures of the neurocranium have necessarily developed from cartilaginous precursors is not unreasonable, permitting application of conservative, cartilaginous nomenclature to relatively derived osteological structures.

**Fig 1:**
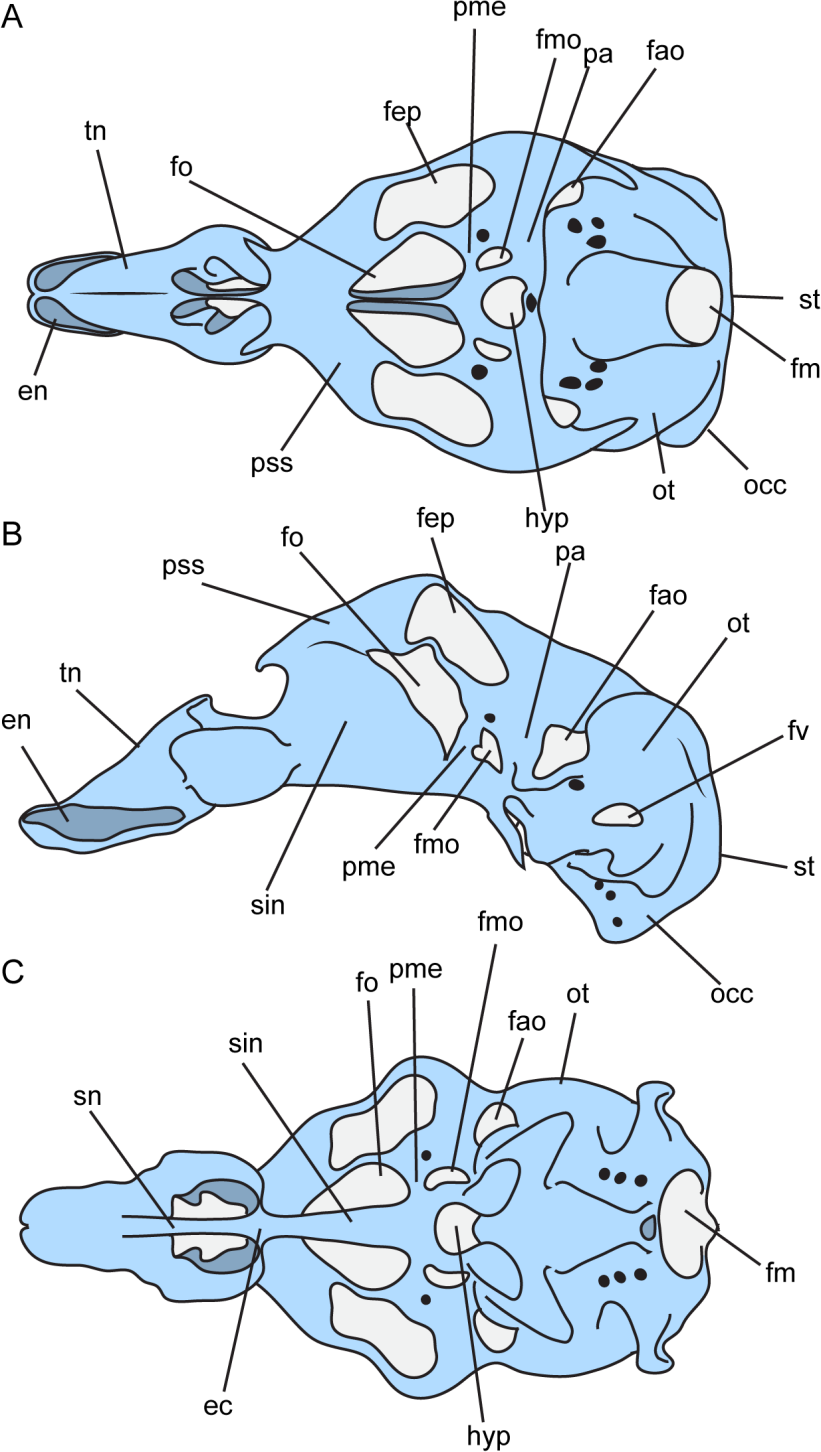
Embryonic morphology of the cartilaginous neurocranium of a generalized tetrapod, *Crocodylus porosus*, after [26]. **A**, dorsal view; **B**, lateral view; **C**, ventral view. Illustrations not to scale. **Abbreviations**: **ec**, ethmoid commissure; **en**, external naris; **fao**, foramen antoticum; **fep**, fenestra epioptica; **fm**, foramen magnum; **fmo**, foramen metopticum; **fo**, foramen ovale; **fv**, fenestra vestibularis; **hyp**, hypophyseal fenestra; **occ**, occipital arch; **ot**, otic capsule; **pa**, pila antotica; **pme**, pila metoptica; **pss**, planum supraseptale; **sin**, interorbital septum; **sn**, nasal septum; **st**, synotic tectum; **tn**, tectum nasi.

As this is a nonconventional approach to neurocranial osteology, we provide here a reference of generalized tetrapod neurocranial morphology (Fig 1) with focus on the nomenclature and morphology described in *Quasicaecilia* below.

## Results

### Systematic Paleontology

Lepospondyli Zittel 1888

Recumbirostra Anderson 2007

Brachystelechidae Carroll & Gaskill 1978

*Quasicaecilia texana* Carroll 1990

(Figs 2, 3 & 4)

**Fig 2:**
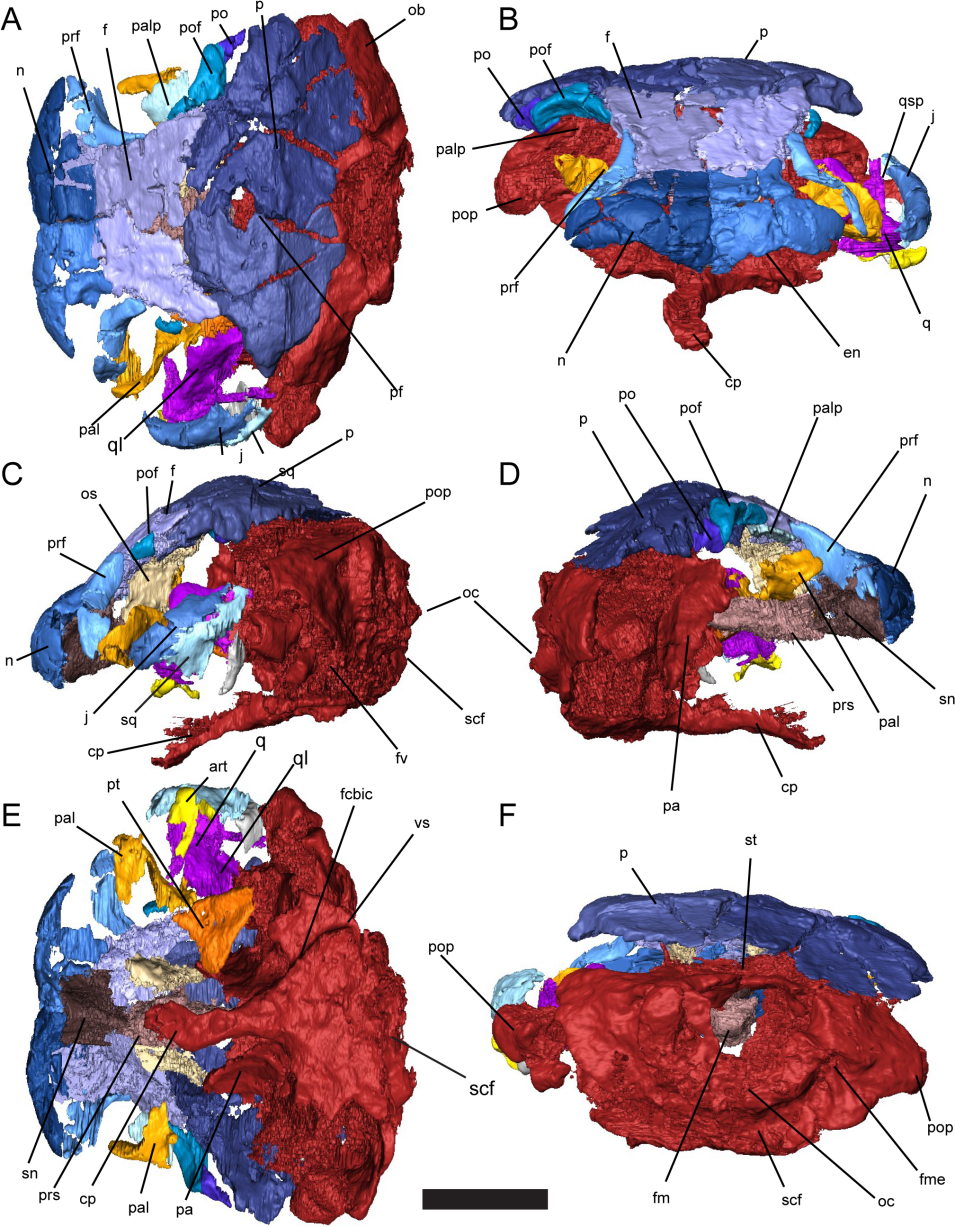
Skull of *Quasicaecilia texana*, USNM 22079, 3D renders segmented from μCT. **A**, dorsal view; **B**, anterior view; **C**, left lateral view; **D**, right lateral view; **E**, palatal view; **F**, occipital view. Scale bar equals 5 mm. Abbreviations: **cp**, cultriform process; **en**, dorsal margin of external naris; **f**, frontal; **fcbic**, foramen serving cerebral branch of the internal carotid artery; **fm**, foramen magnum; **fme**, foramen metoticum; **fv**, fenestra vestibularis; **j**, jugal; **n**, nasal; **oc**, occipital condyle; **os**, orbitosphenoid; **p**, parietal; **pa**, pila antotica; **pal**, palatine; **palp**, palpebral bone; **pf**, pineal foramen; **po**, postorbital; **pof**, postfrontal, **pop**, paroccipital process; **prf**, prefrontal; **prs**, presphenoid; **pt**, pterygoid; **q**, quadrate; **qsp**, stapedial process of quadrate; **scf**, subcondylar fossa; **sn**, nasal septum; **sq**, squamosal.

**Fig 3:**
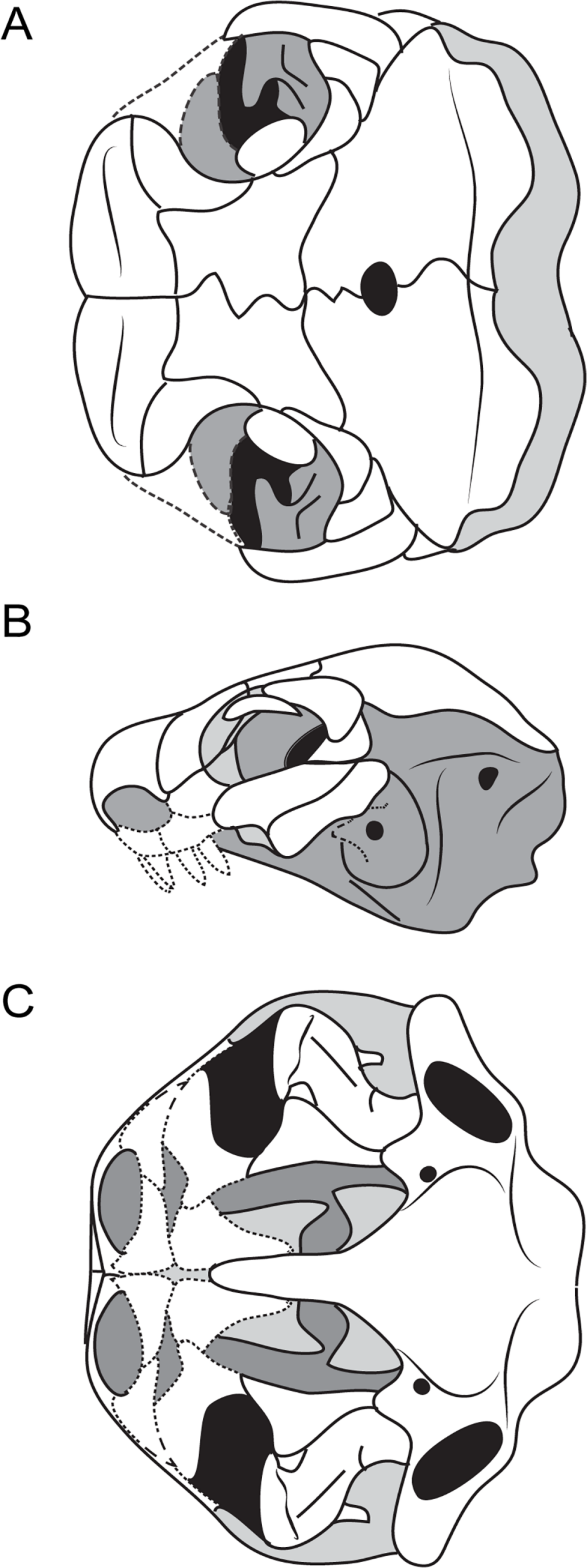
Reconstruction of skull of *Quasicaecilia texana*. **A**, dorsal view; **B**, left lateral view; **C**, ventral view. Dashed lines indicate inferred or hypothetical morphology.

**Fig 4:**
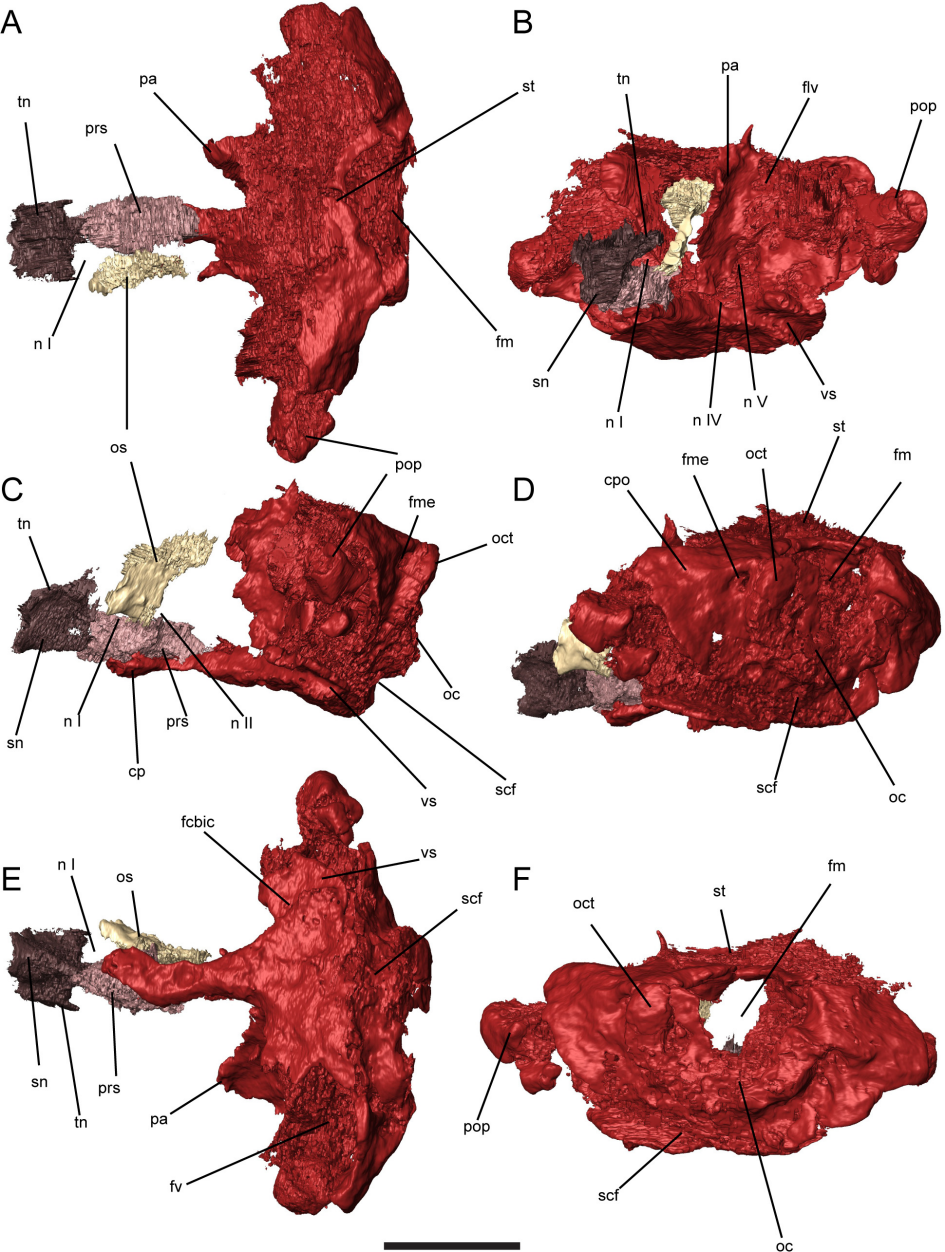
Braincase of *Quasicaecilia texana*, USNM 22079, 3D renders segmented from μCT. Anterior and posterior braincase digitally re-assembled. **A**, dorsal view; **B**, left anterolateral view; **C**, left lateral view; **D**, left posterolateral view; **E**, ventral view; **F**, posterior view. Scale bar equals 5 mm. Abbreviations: **cp**, cultriform process; **fcbic**, foramen serving cerebral branch of the internal carotid artery; **flv**, foramen serving lateral head vein; **fm**, foramen magnum; **fme**, foramen metoticum; **fv**, fenestra vestibularis; **n I**, foramen serving olfactory nerve; **n II**, foramen serving optic nerve; **n IV**, foramen serving trochlear nerve; **n V**, foramen serving trigeminal nerve (undivided); **oc**, occipital condyle; **oct**, occipital cotyle; **os**, orbitosphenoid; **pa**, pila antotica; **pop**, paroccipital process; **prs**, presphenoid; **scf**, subcondylar fossa; **sn**, nasal septum; **st**, tectum synoticum; **tn**, tectum nasi; **vs**, vidian sulcus.

#### Holotype

Isolated skull, USNM 22079

#### Locality and Horizon

The type locality of *Quasicaecilia texana* is uncertain. The type and only specimen was collected from the early Permian redbeds of Texas by Charles H. Sternberg, but no precise locality information was recorded. Carroll [23] suggested that this specimen was likely from the Arroyo Formation (Clear Fork Group) of Baylor County, Texas, constraining the age to the Artinskian (290–279 Ma).

#### Revised Diagnosis

Brachystelechid with the following autapomorphies: nasal pair very wide anteriorly, unique ossification within the nasal septum and tectum nasi separate from ossification of interorbital septum, external nares anteroventrally-directed, occipital cotyles at level of the dorsal extent of foramen magnum, prominent fossa for muscle attachment ventral to the occipital condyle on occipital surface.

### Description

#### General

Carroll [23] previously described the external morphology of the skull of *Quasicaecilia texana*, but our new data resolve some uncertainties in the initial description. Moreover, the recent redescription of the skull of the brachystelechid *Carrolla craddocki* [9] serves as a ready comparison for the morphology of *Quasicaecilia texana*, permitting more robust inferences of the homology of bones and of soft tissue anatomy. The skull roof and braincase are essentially complete (Fig 2), but most of the lower portion of the skull is missing, including the premaxilla and maxilla, cheek, and most of the palate, as well as both lower jaws. The skull is mostly articulated, undistorted, and preserved in three dimensions, although the posterior braincase unit is partially dissociated from the remainder of the skull and displaced posteroventrally.

The bone is generally well preserved, although dermal elements show substantial fracturing. A large crack, previously interpreted to be the frontal-nasal suture [23], passes transversely through the front of the skull. In the micro-CT data, the bone and matrix show differences in radiocontrast, although portions of the skull are obscured by high iron oxide content of the matrix, which impacts on the overall detail that can be gleaned from the scans. Numerous centres of iron precipitate within the matrix and within pore space of the bone also serve to obscure fine detail in certain parts of the skull. As a result, some fine details of morphology are less clear in *Quasicaecilia texana* than *Carrolla craddocki* [9].

The skull of *Quasicaecilia texana* is short, broad, and strongly recumbent (Figs 2 & 3). The anterior margin of the skull roof flares laterally to form a broad, shovel-like snout. The dorsally-facing orbits are very large, with a diameter nearly 50% of the length of the skull (Figs 2A & 3A). The ant- and postorbital regions are both very short, encompassing no more than 20% and 30% of the total skull length, respectively. The front half of the skull is steeply downturned (Figs 2C and 2D & 3B), with the ethmoid region of the skull extending ventral to the plane of the basioccipital (Fig 4B). The external nares are situated at the anterior edge of the snout, and open ventrally (Fig 2B).

A single flat palpebral ossicle is present above the orbit, as in *Huskerpeton englehorni* [22] and *Carrolla craddocki* [9]. Although palpebral ossicles have been reported in dissorophoids [27–32], these occur as a mosaic of many thin, tiny elements and thus differ from the recumbirostran condition.

#### Skull roof and cheek

The skull roof and cheek are comprised of a relatively reduced complement of bones in comparison with other recumbirostrans. The skull roof consists of nasals, frontals, parietals, prefrontals, and postfrontals (Fig 2A). The cheek is strongly anteriorly-canted, and is comprised of a jugal and a squamosal, the latter of which may be fused with the quadratojugal (Fig 2C). The premaxilla and maxilla are missing, as are the lacrimal and postorbital. It is unclear the degree to which the absence of the latter two elements is taphonomic, or if some are a true loss of these bones.

The nasals are widest along the anterior margin of the snout and taper posteriorly. The anterior portion of the nasals curves ventrally to meet the dorsal margin of the external nares. The nasals are buttressed internally by a median septum that fits tightly into a groove in the anterior braincase. This median septum apparently served as the only contact between the nasal and premaxilla; the nares are otherwise narrowly spaced and the nasals show no evidence of an additional contact for the pars dorsalis of the premaxilla. A large crack running transversely across the anterior skull has been interpreted previously as the frontal-nasal suture, which would restrict the nasal to the very anterior margin of the skull [23]. The micro-CT data in parasagittal section shows that the nasal actually extends far posterior to this crack and overlaps the frontal to form a broad scarf joint at the level of the orbit. The nasals articulate with the prefrontal, but the extent of this articulation is unclear, as both prefrontals are partially damaged. It seems unlikely, however, that there would have been extensive contact between the nasal and lacrimal.

The frontals are roughly rectangular, with a lateral expansion posteriorly where the frontal contributes to the dorsal margin of the orbit. The median suture between the frontals is deeply sinusoidal. A series of four to five large pits of subequal size is present on the dorsal surface of the frontals parallel to the orbital margin. Similar pitting is present parallel to the dorsal orbital margin in the brachystelechid *Batropetes fristchi*, where it occurs on the frontals, [24, 25] and in the lysorophian *Brachydectes newberryi*, where it occurs on the prefrontals (Pardo, pers. obs.). The internal surface of each frontal sends a low, robust flange ventrally to meet the orbitosphenoid.

The parietal pair dominates the posterior portion of the skull roof. Broad occipital lappets of the parietals connect the skull roof to the underlying otoccipital compound ossification. A wide pineal foramen is present and passes through the anterior half of the parietal pair. Paired ventral flanges of the parietals meet the ventral flanges of the frontals and contact the posterior end of the orbitosphenoids as well as the ossifications of the pila antotica.

The prefrontal is a relatively small, falciform element in the anterior orbit. The prefrontal is incomplete and appears to be excluded from the external naris by a lacrimal-nasal contact. A well-developed dorsal process contacts the ventral flange of the frontal but does not contribute towards a solid antorbital wall as seen in *Nannaroter mckinziei* [22] and *Rhynchonkos stovalli* [10], nor does it form a deep notch to enclose the deep ophthalmic nerve as seen in these taxa. The relationship between the prefrontal and lacrimal is unclear; the separate ossification previously identified as a disarticulated lacrimal [23] is interpreted here as a palatine based on the presence of the choanal margin. No nasolacrimal foramina are present along the antorbital margin of the prefrontal, indicating that it has not expanded to incorporate the lacrimal bone. Whether an independent lacrimal was present, or whether this element has fused with the maxilla, is unclear.

The postfrontal is a small, falciform element posterior to the orbit. Posteriorly, it is completely excluded from the temporal region by the extremely wide parietals. The postfrontal does not meet the prefrontal dorsally. Laterally, the postfrontal weakly overlaps a small fragment of bone that may represent the remnant of the postorbital, but this element is not well-preserved enough to confidently identify its homology.

The jugal is a narrow, tall element that forms the posterior margin of the orbit. The jugal is anteriorly-canted and very high, extending approximately half the height of the orbit. Beneath the orbit, there is a weak medial flange of the jugal possibly equivalent to the alary process of the jugal described in some early tetrapods and amniotes.

Posterior to the jugal, the posterior margin of the cheek is comprised of a single elongate, anteriorly-canted element here interpreted as either the squamosal or fusion of the squamosal and quadratojugal. An occipital flange of the squamosal is present. The quadrate fits tightly into a medially located trough in the ventral portion of this bone.

The dorsal part of the temporal region shows a reduced complement of dermatocranial elements, resulting in a substantial exposure of the dorsal surface of the otic capsules on the skull roof (Fig 3B). In many recumbirostrans, the otic and occipital regions extend posterior to the dermatocranial components of the skull roof and cheek to some degree. In the bracystelechid *Carrolla craddocki*, and apparently in *Quasicaecilia texana*, this pattern is exaggerated with the loss of the postparietals and the temporal series [9]. In *Quasicaecilia texana*, the occipital lappets of the parietals are withdrawn anteriorly compared to the condition in *Carrolla craddocki*, and the cheek is steeply canted anteriorly, exposing much of the otic capsule laterally.

#### Palate

The palate of *Quasicaecilia texana* is largely incomplete. The quadrate is well-preserved, but the pterygoid and palatine are incomplete, and the vomers are missing. There is no evidence of an ectopterygoid or epipterygoid.

The quadrate is the best-preserved element of the palate and suspensorium. The body of the quadrate is antero-posteriorly compressed, and flares dorsally to brace against a prominent ridge on the anterior portion of the otic capsule. A strongly-developed stapedial process extends posteriorly. As in other microsaurs [9, 10, 22] and amniotes [33], the articular surface of the quadrate is comprised of two prominent condylar surfaces. The anterior surface of the quadrate is marked by a sizable fossa just dorsal to the condylar surface.

The pterygoid is small and triangular in palatal view. The quadrate process is short and lacks a well-developed dorsal lamina. The palatine ramus is incompletely preserved, but appears to be greatly reduced to a single narrow strut oriented towards the palatine. No transverse flange is present.

The epipterygoids are absent. It is possible that the epipterygoid has simply been disarticulated from the rest of the skull. However, it is also possible that the epipterygoid was present but cartilaginous in the holotype specimen. In the lysorophian *Brachydectes newberryi*, the epipterygoid ossifies very late in comparison with the remainder of the skull (Pardo & Anderson, pers. obs.), suggesting that ossification of the epipterygoid may be an indicator of skeletal maturity. If so, it is possible that the holotype specimen of *Quasicaecilia texana* represents a juvenile or subadult animal. It is also possible that the lack of an ossified epipterygoid reflects element-specific paedomorphosis in *Quasicaecilia*.

The right and left palatines are poorly-preserved, making inference of the shape of this element difficult. The element appears to have been relatively broad, possibly with a tooth or row of teeth, but the relationship with the remainder of the palate is unclear. A dorsal lamina of the palatine appears to form the anteroventral wall of the orbit, but whether this contributed to a solid antorbital wall such as that seen in *Nannaroter mckinziei* [34] is uncertain, as the lacrimal is not preserved. The margin of the choana is completely absent from the preserved fragments as well.

#### Neurocranium

The braincase of *Quasicaecilia texana* is extensively ossified and mostly complete, although the posterior braincase unit is displaced ventrally from the remainder of the skull. The braincase is narrow in the ethmoid and sphenoid regions, and the otoccipital region is nearly three times as wide as the sphenoid region.

The base of the anterior braincase unit is made up of a robust median ossification, the equivalent to the median bone described in *Carrolla craddocki* [9] and the median anterior bone in *Rhynchonkos stovalli* [10], but here identified as a presphenoid (see Discussion). This element extends from the ethmoid commissure to the optic foramen and supports the orbitosphenoids with conspicuous lateral buttresses. The presphenoid of *Quasicaecilia* is very narrow and deep, forming an interorbital septum.

A second median ossification is present in the ethmoid region anterior to ethmoid commissure and encompassing the columella ethmoidalis, nasal septum, and tectum nasi. The nasal septum extends anteriorly to brace directly against the spade-like anterior margin of the nasals, with a median groove to accept the median nasal buttress. The tectum nasi directly underplates the nasal pair across the majority of their width, further reinforcing the anterior skull against dorsoventral compression.

The left orbitosphenoid is well-preserved. The orbitosphenoid pair is narrow at the base but widens dorsally, forming a deep V-shape in cross section. Dorsally, the orbitosphenoids articulate broadly with a set of descending flanges of the frontal and parietal as in other recumbirostrans [22] and in captorhinids [33]. Although the orbitosphenoid is emarginated posteriorly, presumably around the optic and oculomotor nerve foramina, no distinct foramina are preserved, nor is there a clear incisure in the posterior margin of the orbitosphenoid as is seen in *Carrolla* and *Brachydectes*.

The posterior braincase consists of a single co-ossified complex encompassing the parasphenoid, sella turcica, pilae antoticae, otic capsules, and occipital arch forming an otoccipital bone. A similar massively co-ossified element has been described in *Carrolla craddocki* [9].The cultriform process connects the otoccipital region of the otoccipital bone with the anterior elements of the braincase. The cultriform process is itself narrow and parallel-sided with a dorsal groove to accept the presphenoid. The braincase is highest at the point of the pila antotica, which are antero-posteriorly shortened, and articulates dorsally with a ventral flange of the parietals. In transverse section, ossifications of the pila antotica flare laterally to form a narrow, deep V-shaped cavum cranii. Three foramina pass through the region between the pila antotica and the otic capsule: a large medioventral foramen serving the trigeminal nerve, a small far-ventral foramen serving the trochlear nerve, and a small dorsal foramen potentially serving a branch of the lateral head vein (Fig 4B).

The base of the otoccipital bone is greatly laterally expanded but anteroposteriorly shortened in ventral view (Fig 4E). The vidian sulcus, which indicates the course of the common internal carotid artery, passes along the basal plate of the braincase medial to the basipterygoid processes. A foramen pierces the basal plate at approximately the level of the basipterygoid processes, likely representing the course of the cerebral branch of the internal carotid artery. The vidian sulcus continues anteriorly, serving as the course of the palatine branch of the internal carotid. There are grooves along the lateral edge of the cultriform process in some recumbirostrans that indicate the anterior course of the palatine branch of the internal carotid artery, but they are absent in *Q*. *texana*.

The otoccipital region of the skull posterior to the pila antotica is extremely high and anteroposteriorly shortened in lateral view (Fig 4C). The otic capsules are fully co-ossified without evidence of a separate prootic or opisthotic. The synotic tectum is fully ossified, bridging the otic capsules dorsally (Fig 4A), but lacks the sagittal ridge seen in some other recumbirostrans. The anteroventrally oriented fenestra vestibularis is large and oval. A few fragments of bone can be seen within the left fenestra vestibularis, but whether these represent remnants of the stapes (as previously reported [23]) or dissociated elements forced into the fenestra vestibularis cannot be determined. Anterior to the fenestra vestibularis is a dorsoventral ridge of bone that appears to articulate with the quadrate. Dorsal to the fenestra vestibularis is a weakly developed crista parotica, which extends anterolaterally into a robust paroccipital process. The synotic tectum is straight anteriorly, without conspicuous median or lateral ascending processes.

The occipital region is foreshortened and laterally expanded compared with other recumbirostrans. The foramen magnum is large and restricted to the dorsal half of the occipital surface. It is essentially round in shape, rather than diamond-shaped as in *Carrolla craddocki* [9]. As in other recumbirostrans, the occipital contribution to the atlantoccipital joint consists of a shelflike cotylar surface of the basioccipital and paired exoccipital condyles. In contrast to other recumbirostrans, the exoccipital condyles are situated dorsolateral to, rather than ventral to, the foramen magnum, potentially allowing for greater involvement of the hypaxial musculature during depression of the head. The foramen metoticum is located just anterior to the exoccipital condyle. Whether a separate hypoglossal foramen was present is unclear. Ventral to the occipital condyle is a wide fossa, termed here the subcondylar fossa (Fig 4D). This may represent a muscle attachment site homologous with the basal tubera of many early tetrapods based on location and general conservativism of the axial musculature in tetrapods, but no distinct tubera are apparent. This fossa may have accommodated insertion of the m. longissimus capitis pars transversalis cervicus, the m. iiliocostalis capitis, and/or the m. longus colli, a set of muscles all associated with depression of the head in reptiles [35].

## Discussion

### Homology of Anterior Braincase Bones in Recumbirostrans

**Fig 5:**
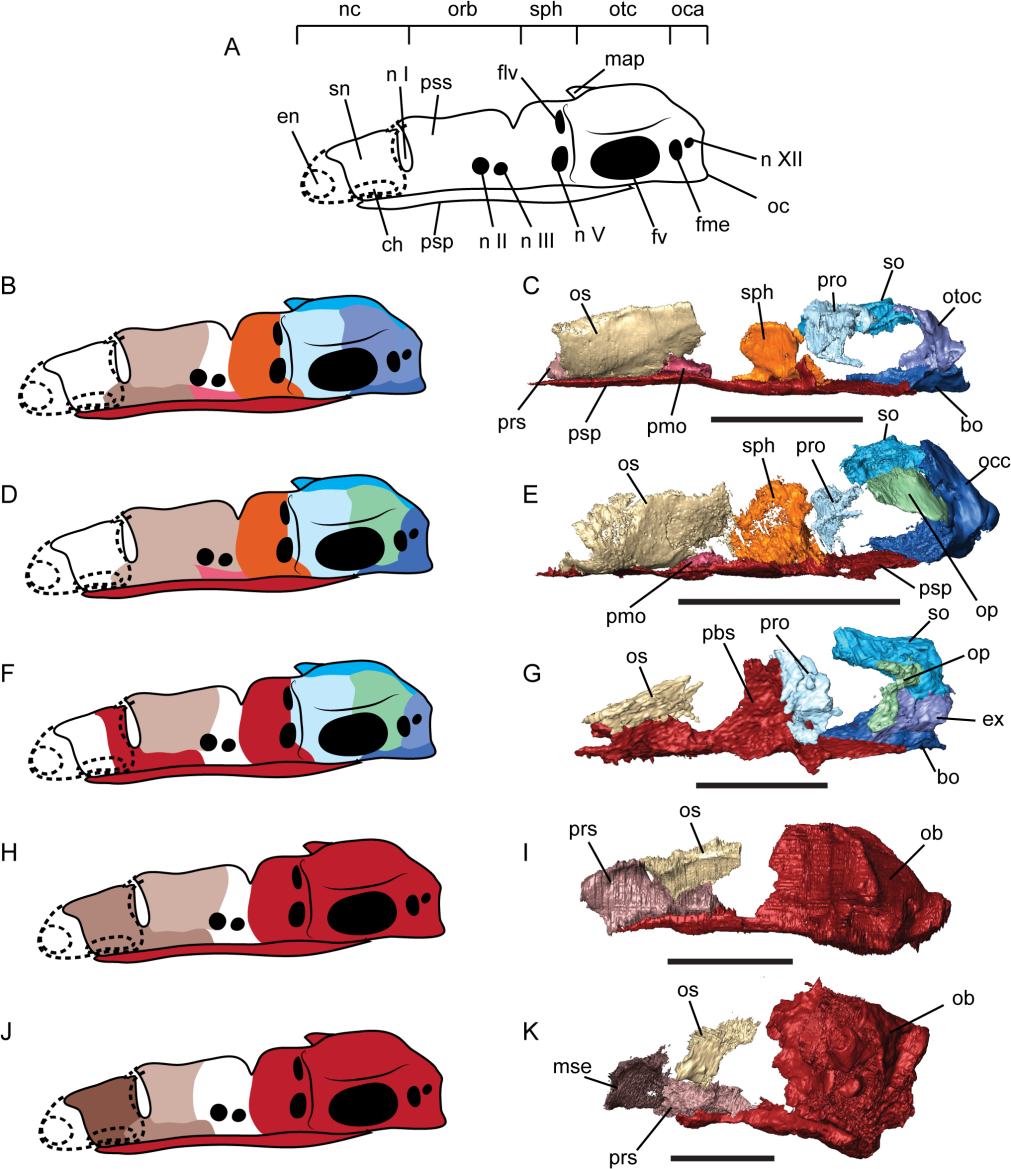
Arrangement of braincase ossifications in selected Recumbirostra. **A**, diagrammatic illustration of structures and key landmarks in the recumbirostran braincase. **B**, map of ossified regions in the braincase of *Huskerpeton englehorni*. **C**, digital dissection of braincase of *Huskerpeton englehorni*, UNSM 32144; **D**, map of ossified regions in the braincase of *Dvellecanus carrolli*, UCMP 202940; **E**, digital dissection of braincase of *Dvellecanus carrolli*, UCMP 202940; **F**, map of ossified regions in the braincase of *Brachydectes newberryi*; **G**, digital dissection of braincase of *Brachydectes newberryi* (KUVP 49541); **H**, map of ossified regions in the braincase of *Carrolla craddocki*; **H**, digital dissection of braincase of *Carrolla craddocki* (TMM 40031–54), modified from [9]; **I**, map of ossified regions in the braincase of *Quasicaecilia texana*, **J**, digital dissection of braincase of *Quasicaecilia texana*, USNM 22079. Scale bars equal 5 mm. Abbreviations: **bo**, basioccipital; **ch**, choana; **en**, external naris; **ex**, exoccipital; **flv**, foramen serving the lateral head vein; **fme**, foramen metoticum; **fv**, fenestra vestibularis; **map**, median ascending process of the synotic tectum; **mse**, mesethmoid; **nc**, nasal capsule; **n I**, foramen serving the olfactory nerve; **n II**, optic foramen; **n III**, oculomotor nerve foramen; **n V**, trigeminal nerve foramen; **n XII**, hypoglossal foramen; **ob**, otoccipital bone; **oc**, occipital condyle; **oca**, occipital arch; **occ**, occipital; **op**, opisthotic; **orb**, orbital cartilages; **os**, orbitosphenoid; **otc**, otic capsule; **otoc**, otoccipital; **pbs**, parabasisphenoid; **pmo**, posterior median ossification of the ethmoid trabeculae; **pro**, prootic; **prs**, presphenoid; **psp**, parasphenoid; **pss**, planum supraseptale; **sph**, sphenoid; **so**, supraoccipital.

The high level of ossification in the recumbirostran braincase has led to some discussion of the homologies of the elements observed [9, 22]. Of particular interest are conserved centers of ossification within the braincase that have been interpreted as neomorphs arising within Recumbirostra, including the orbitosphenoid [9], and presphenoid [9]. Many of these elements occupy a similar topological position as ossifications within the braincase of early amniotes [34, 36], but homologies between the amniote elements and the recumbirostran counterparts have generally been rejected given the apparent absence of these ossifications in other ‘lepospondyls’ [21, 22, 36, 37] and in some cases on positional criteria [9]. This has been complicated by simplification of the braincase in many recumbirostrans both by the consolidation of individual elements into large compound elements [9] and the overall reshaping of the braincase in association with miniaturization [9, 23]. We present here a generalized schema of the recumbirostran braincase (Fig 5A) based on our μCT based survey of recumbirostran skull morphology in order to approach this homology problem.

In most recumbirostrans, the anterior braincase consists of paired lateral elements within the planum supraseptale and a median unpaired element within the trabecula communis and columella ethmoidalis. The homologies of these elements have been discussed in some detail, but extensive comparative material has largely been lacking [9, 10, 21, 22, 34].

The paired lateral element of the recumbirostran anterior braincase has been homologized with the sphenethmoid (21, 22, 34) and orbitosphenoid [9]. The paired lateral element encompasses the region of the planum supraseptale (= preoptic root) from its anterior extent posterior to the foramen ovale. In conservative recumbirostrans like *Huskerpeton englehorni* (Fig 5B and 5C), this ossification terminates at the level of the foramen ovale, with no ossification of the pila metoptica. This ossification does invade the pila metoptica in some derived recumbirostrans, including *Dvellecanus carrolli* (Fig 5D and 5E), and the ostodolepids *Nannaroter mckinziei* [34] and *Pelodosotis elongatum* [21]. Although these elements have generally been referred to as sphenethmoids [21, 22, 34], this term generally refers to a compound bone incorporating the entire orbital cartilage unit and posterior nasal capsule [38].

More recently, the paired lateral element of the anterior braincase of *Carrolla craddocki* has been termed the orbitosphenoid [9] based on its position within the preoptic root, and has been homologized with the orbitosphenoid of anurans and caudates, but not the orbitosphenoid of reptiles, which originates from an ossification center within the pila metoptica [38]. However, this is not universally the case; ossification begins within the orbital cartilage in the platypus, *Ornithorhynchus* [38], and in the chicken, *Gallus*, the orbitosphenoid forms from two ossification centers; one in the preoptic root of the orbital cartilage and one within the pila metoptica [38]. Additionally, the planum supraseptale is heavily ossified in early synapsids [39] and in the early reptile *Captorhinus laticeps* [33], but no ossification of the pila metoptica is present in these taxa. The ossification center of the orbitosphenoid in modern salamanders also varies in its location, with ossification initiating within the center of the lateral wall of the braincase in hynobiids and ambystomatids, but initiating dorsal to the foramen ovale in the salamandrid *Triturus* [40]. We follow the usage of Maddin et al. [9] here in referring to these paired bones as orbitosphenoids, with the caveat that this applies also to ossifications of the orbital cartilages in most early tetrapods, including early amniotes.

The median ventral element of the recumbirostran braincase appears to be derived from the trabeculi communis based on position and similar contribution to the interorbital septum. This element is flat and lozenge-shaped in *Huskerpeton* (Fig 5B and 5C) and *Brachydectes* (Fig 5F and 5G), and is expanded into an interorbital septum in *Carrolla* (Fig 5H and 5I) and *Quasicaecilia* (Fig 5J and 5K). Such ossifications are termed presphenoids when they appear in modern amniotes [38] and in some early synapsids [39], and this terminology is used here. It should be noted that in *Dvellecanus carrolli*, the presphenoid is not present and has replaced by median extensions of the orbitosphenoids (Fig 5D and 5E). The presphenoid of the lysorophian *Brachydectes newberryi* appears to have fused to the underlying parasphenoid (Fig 5F and 5G).

A single anterior ossification of the nasal septum and tectum nasi is seen in *Quasicaecilia* (Fig 5J and 5K). Ossifications of the nasal septum are generally termed the mesethmoid in modern tetrapods [38], which we follow here. We note that a fully-ossified nasal septum analogous or possibly homologous to the condition in *Quasicaecilia texana* has been reported in a very large specimen of *Captorhinus laticeps* [33], but in this taxon, as in *Carrolla craddocki*, a single element encompasses the interorbital septum, nasal septum, and columella ethmoidalis and no separate mesethmoid is present.

A second median ossification posterior to the presphenoid is present below the optic and oculomotor foramina in *Huskerpeton englehorni* (Fig 5B and 5C) and in *Dvellecanus carrolli* (Fig 5D and 5E). This element is distinct from the presphenoid and orbitosphenoids and may represent the anterior margin of the hypophyseal fossa.

### Patterns of Neurocranial Consolidation in Recumbirostrans

Consolidation of neurocranial bones into a few extensively-ossified compound elements is seen repeatedly in fossorial tetrapods such as amphisbaenids, caecilians, and uropeltid snakes [12, 13, 15–19]. A similar trend is observed within recumbirostrans, and is achieved in a variety of ways.

Consolidation of the occipital region is first seen in conservative recumbirostrans. *Huskerpeton englehorni* (Fig 5B and 5C) shows co-ossification of the exoccipital and opisthotic into a single compound element. This morphology has previously been reported in *Rhynchonkos stovalli* [21] but subsequent restudy of this material shows that the exoccipital and opisthotics are distinct elements with a sutural contact passing though the foramen metoticum [10]. In *Dvellecanus carrolli*, the exoccipitals are fully co-ossified with the basioccipital (Fig 5D and 5E) instead.

More extensive consolidation of the braincase is seen in morphologically-derived recumbirostrans. The lysorophian *Brachydectes newberryi* has an elongate, zygokrotaphic skull approaching the morphology of some amphisbaenids [41, 42]. The parasphenoid in this species is well integrated into the braincase, fusing with the pila antotica as well as the presphenoid, forming a broad, robust parabasisphenoid (Fig 5F and 5G).

The brachystelechids *Carrolla craddocki* (Fig 5H and 5I) and *Quasicaecilia texana* (Fig 5J and 5K) show the most extensive consolidation of the braincase. The pila antotica, parasphenoid, otic region, and occiput are all fully co-ossified into a single massive otoccipital complex. A similar condition is seen in a number of modern fossorial lineages, including caecilians (16–19), uropeltid snakes [15], and amphisbaenians [13]. Interestingly, *C*. *craddocki* and *Q*. *texana* show expansion of the anterior ossifications of the braincase as well; the presphenoid in these taxa expands far anterior, encompassing the entire septum nasale in *C*. *craddocki*, and meeting a separate mesethmoid element in *Q*. *texana*. In the latter case, the mesethmoid has expanded dorsally to encompass much of the tectum nasi as well as the nasal septum.

### Arrangement of Neck Musculature in Recumbirostrans

Although previous studies have noted that expanded attachment sites for the epaxial musculature may be evidence of recumbirostran fossoriality [9, 22], little to no attention has been given to the ventral musculature of the neck in recumbirostrans. The discovery of a well-developed fossa for attachment of the deep musculature of the ventral neck is a first for a recumbirostran, and suggests that adaptations within the ventral muscle groups of the neck, such as the m. iliocostalis and m. longus groups, may have been overlooked. In *Quasicaecilia*, this is also coupled with a marked dorsal displacement of the occipital condyles, which provides better mechanical advantage during cranial depression and accommodates additional expansion of the hypaxial musculature of the neck. In some modern amphisbaenians, the excavatory cycle is a three-step (during descent) or four-step (during ascent) process [43, 44]. Depression of the skull plays an important role in this process, allowing the animal to penetrate its snout into the soil prior to the upstroke, thus increasing the amount of soil displaced [14, 43, 44]. This behavior allows modern amphisbaenians to excavate extensive subterranean galleries in compact soil, a specialized condition even within fossorial reptiles. The expansion of the ventral musculature in *Quasicaecilia texana* suggests that some recumbirostrans may have evolved an excavatory cycle similar to that of shovel-snouted amphisbaenians and thus may have had similar fossorial capabilities.
